# Dr. Wells’ Essays on Dysentery, Diarrhœa, Typhoid Fever, Etc.

**Published:** 1883-01

**Authors:** 


### DR. WELLS' ESSAYS ON DYSENTERY, DIARRHŒA, TYPHOID FEVER, ETC., ETC.

We commence in this issue of THE HOMŒOPATHIC PHYSICIAN to publish a series of incomparable essays on the treatment of dysentery, diarrhœa, typhoid fever, rheumatism, intermittent fever, etc.—all from the gifted pen of Dr. P. P. Wells.

Some of these essays were published twenty years ago; these will be revised by the author before being republished.

Others are new; the one on dysentery has been entirely re-written.

They will be paged for separate binding, and when completed will make a large and most useful volume.

It is not too much to say that we have not in homœopathic literature any practical essays to compare with these.

